# Fecal virome transplantation attenuates arthritis in mice by remodeling gut ecology, systemic tryptophan metabolism, and innate immune responses

**DOI:** 10.1038/s41522-026-00980-2

**Published:** 2026-04-08

**Authors:** Lun He, Difen Yuan, Qian Li, Xuan Zhang, Kevin Niu, Xin Li, Yanhua Ou, Haoming Du, Jiajia Yuan, Yuanyuan Duan, Haitao Niu

**Affiliations:** 1 Key Laboratory of Viral Pathogenesis and Infection Prevention and Control (Jinan University), Ministry of Education, School of Medicine, Jinan University, Guangzhou, China; 2 Guangzhou Key Laboratory for Germ-Free Animals and Microbiota Application, Institute of Laboratory Animal Sciences, Jinan University, Guangzhou, China; 3 School of Basic Medicine, Yunnan University of Chinese Medicine, Kunming, China; 4 Sargent College of Health and Rehabilitation Sciences, Boston University, Boston, MA USA

**Keywords:** Diseases, Immunology, Microbiology

## Abstract

Rheumatoid arthritis (RA) is an autoimmune disorder characterized by chronic joint inflammation and systemic immune dysregulation. Emerging evidence suggests that the gut microbiome plays an important role in immune modulation in RA, yet the role of the gut virome remains poorly understood. Here, using the K/BxN serum-transfer arthritis model, we systematically evaluated the potential role of fecal virome transplantation (FVT) in modulating gut ecology and innate inflammatory responses. Arthritic mice exhibited marked alterations in gut virome composition compared with healthy controls. Administration of purified virus-like particles (VLPs) from healthy donors correlated with reductions in paw swelling, histopathological inflammation, bone erosion, circulating proinflammatory cytokines, and myeloid cell infiltration in inflamed tissues. In parallel, 16S rRNA sequencing showed that FVT remodeled the gut bacterial community toward a composition more similar to that of healthy controls. Targeted serum metabolomics revealed increased levels of microbiota-derived tryptophan metabolites, including indole-3-lactic acid and related indole derivatives, suggesting a link between gut microbial remodeling and systemic immunometabolic regulation. Collectively, these findings indicate that FVT may attenuate inflammatory arthritis by remodeling gut microbial ecology, potentially involving virome–bacteriome interactions and immunometabolic pathways.

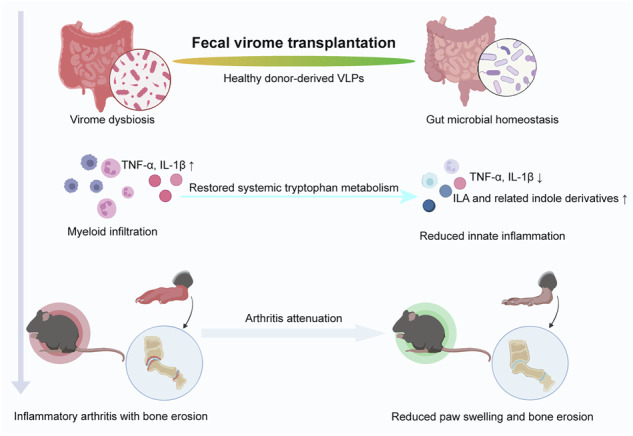

## Introduction

Rheumatoid arthritis (RA) is a chronic inflammatory autoimmune disease characterized by joint inflammation and systemic involvement, imposing a substantial health and socioeconomic burden worldwide^1^. Although current pharmacological therapies can partially control disease activity, they remain limited by suboptimal efficacy and long-term adverse effects^2,3^. Thus, there is an urgent need to develop novel therapeutic strategies.

In recent years, the gut microbiota has been increasingly recognized as a key player in the pathogenesis and immune regulation of RA^4,5^. Numerous studies have demonstrated that intestinal dysbiosis is associated with RA and may influence host immune responses^6,7^. In addition to bacteria, the gut virome—comprising primarily bacteriophages— represents an essential yet often overlooked component of the intestinal ecosystem^8^. Importantly, emerging evidence indicates that the gut phage community is also markedly altered in RA patients, displaying disrupted community structure, depletion of specific phages, and enrichment of auxiliary metabolic genes or molecular mimicry–related peptides potentially linked to autoimmune responses^9–11^. These findings suggest that phages may correlate with RA pathogenesis and highlight the need for further investigation into their functional roles. However, most existing studies are correlative, and the functional role of the gut virome as a therapeutic target remains largely unexplored.

Fecal virome transplantation (FVT), a novel microbiome-based intervention that transfers purified cell-free viral particles, has shown therapeutic potential across multiple disease models^12,13^. Reported benefits include alleviation of intestinal inflammation in inflammatory bowel disease^14,15^, improvement of metabolic abnormalities in obesity and type 2 diabetes^16,17^, promotion of mucosal repair after radiation-induced intestinal injury^18^, and restoration of gut homeostasis in *Clostridioides difficile* infection, where phage-mediated community remodeling achieves efficacy comparable to fecal microbiota transplantation while reducing the risk of pathogen transmission^19,20^. Nevertheless, its application to autoimmune diseases, particularly RA, has not been systematically investigated, and the underlying mechanisms remain elusive.

To directly examine the contribution of the virome to acute inflammatory arthritis, we employed the K/BxN serum-transfer model, which rapidly and reproducibly induces joint inflammation dominated by innate immune cell infiltration following passive transfer of arthritogenic antibodies^21^. This model provides an ideal platform to evaluate the anti-inflammatory effects of FVT. Notably, despite its extensive use in RA research, the dynamic changes of the intestinal virome during this model have not been characterized.

In this study, we performed metavirome sequencing to systematically profile virome alterations in K/BxN serum-induced arthritis, prepared and validated fecal viral particles from healthy donors, and assessed the therapeutic impact of FVT on clinical manifestations, immune cell infiltration, bacterial microbiota composition and tryptophan metabolism. Our findings establish the baseline characteristics of the gut virome in this classical arthritis model and suggest a potential role for gut virome modulation in antibody-mediated inflammatory arthritis.

## Results

### Establishment of the K/BxN serum-transfer arthritis model

**Fig. 1:**
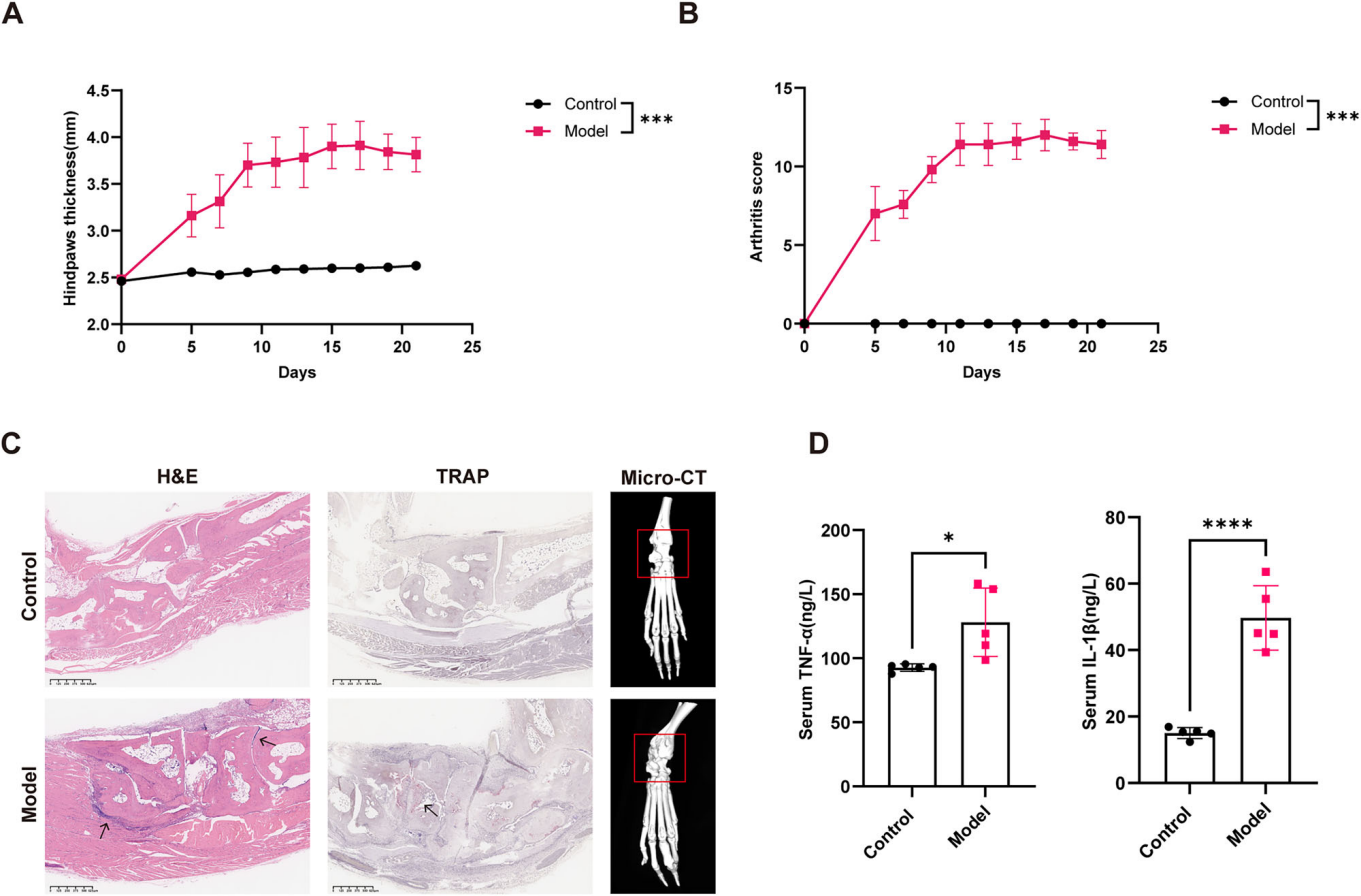
Establishment and validation of the K/BxN serum-transfer arthritis model. **A** Time course of hind paw thickness in the control and serum-transferred groups (*n* = 5 per group). **B** Progression of clinical arthritis scores. **C** Representative histological and imaging results of ankle joints, including H&E staining, TRAP staining, and micro-CT images showing bone erosion. **D** Serum concentrations of TNF-α and IL-1β on day 21. Data are presented as mean ± SD. Statistical comparisons were performed using unpaired two-tailed t-test; *p < 0.05, ***p < 0.001, ****p < 0.0001 compared with the control group.

To establish an arthritis model and examine the early role of the virome, mice were injected intraperitoneally with K/BxN serum once weekly for three consecutive weeks, and samples were collected on day 21. Compared with controls, serum-transferred mice developed rapid and reproducible joint inflammation. Paw swelling became evident in the early phase of the experiment, reached a plateau around day 10, and remained significantly elevated thereafter (Fig. 1A). Clinical arthritis scores increased progressively over time (Fig. 1B). Histological analysis revealed marked synovial hyperplasia, extensive leukocyte infiltration, and cartilage destruction in the model group. TRAP staining demonstrated increased osteoclast activity, and representative micro-CT images showed apparent bone erosion in ankle joints (Fig. 1C). In addition, serum levels of TNF-α and IL-1β were significantly elevated on day 21 compared with controls (Fig. 1D). These findings confirmed that K/BxN serum transfer reliably induces acute inflammatory arthritis and provides a robust foundation for subsequent virome analyses.

### Gut virome alterations in K/BxN serum-transfer arthritis mice

**Fig. 2:**
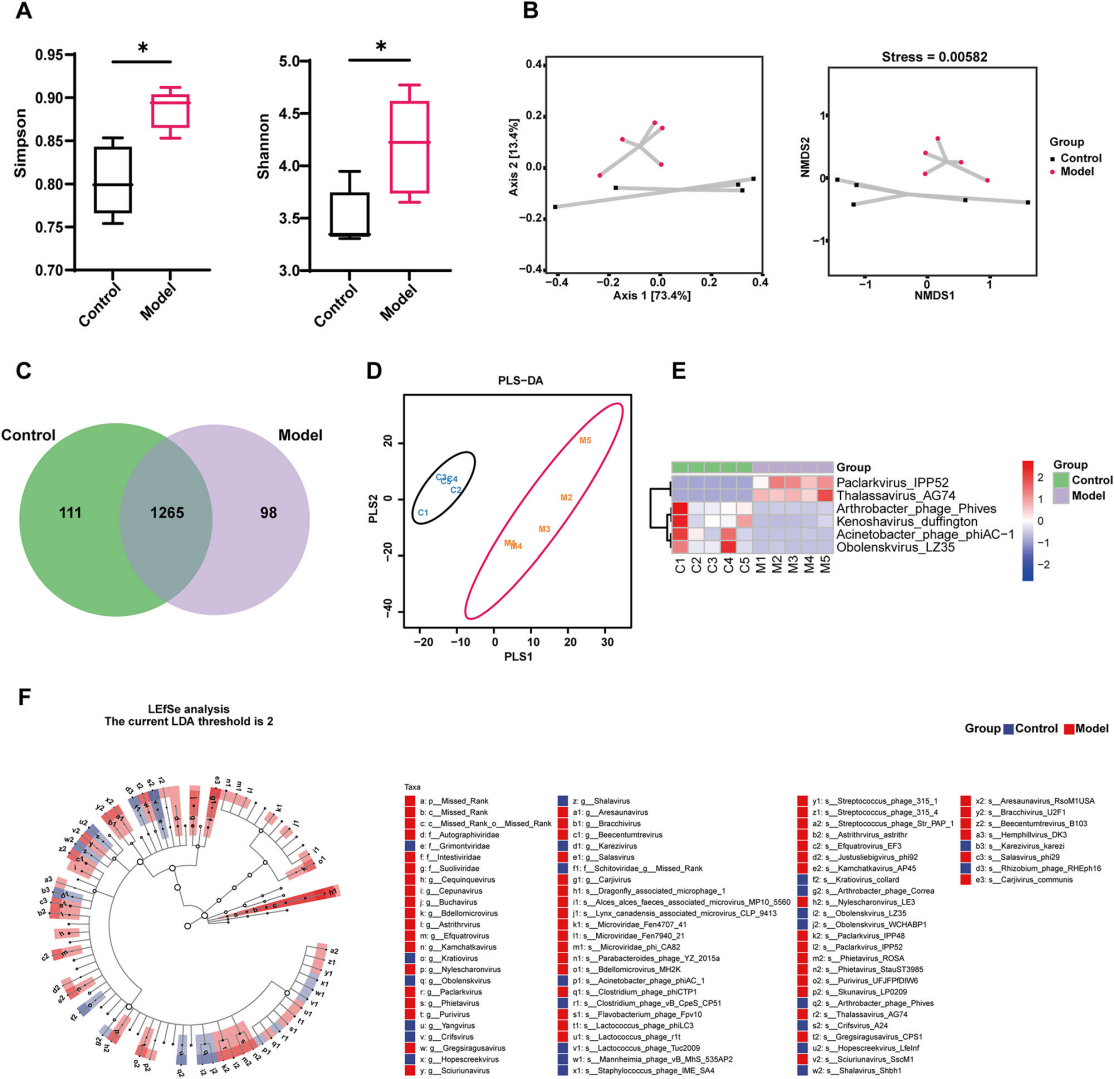
Gut virome alterations in K/BxN serum-transfer arthritis mice. **A** Alpha diversity (Simpson, Shannon; *p* < 0.05; *n* = 5 per group). **B** Beta diversity (PCoA, NMDS; Bray–Curtis), showing separation between groups. **C** Venn diagram of shared and unique vOTUs. **D** PLS-DA of viral community composition showing separation between control and model groups. PLS-DA was used for exploratory visualization. Statistical significance of community differences was assessed using Bray–Curtis distance–based PERMANOVA (999 permutations; *F* = 1.03, R² = 0.114, *p* = 0.018). **E** Heatmap of differentially abundant vOTUs, illustrating group-specific enrichment patterns. **F** LEfSe analysis (LDA > 2.0) showing viral lineages enriched in the model versus control groups.

Metavirome sequencing of fecal virus-like particles revealed significant virome changes in serum-transferred mice. Alpha-diversity indices (Simpson, Shannon) were significantly higher in the model group than in the control group (Fig. 2A). Beta-diversity analyses (PCoA and NMDS based on Bray–Curtis distances) showed clear separation between groups, indicating distinct viral community structures (Fig. 2B).

Venn analysis revealed both shared and unique vOTUs among groups (Fig. 2C). PLS-DA demonstrated visual separation between control (C1–C5) and model (M1–M5) samples and was used for exploratory visualization. Group differences were statistically evaluated using Bray–Curtis distance–based PERMANOVA, which revealed significant compositional differences at the species level (*F* = 1.03, R^2^ = 0.114, *p* = 0.018) (Fig. 2D). The heatmap (row-wise z-scores) revealed a small subset of differentially abundant vOTUs. *Paclarkvirus_IPP52* and *Thalassavirus_AG74* were enriched in the model group, whereas *Arthrobacter_phage_Phives*, *Kenoshavirus_duffington*, *Acinetobacter_phage_phiAC-1*, and *Obolenskvirus_LZ35* were more abundant in the control group (Fig. 2E).

Consistently, LEfSe analysis (LDA > 2.0) identified distinct viral signatures between groups across multiple taxonomic levels (Fig. 2F). In the model group, significant enrichment was observed at the family level for *Autographiviridae, Suoliviridae* and *Intestiviridae*. At finer taxonomic resolution, several genera—including *Paclarkvirus*, *Phietavirus*, *Efquatrovirus*, and *Kamchatkavirus*—were also significantly enriched. In contrast, the control group exhibited enrichment of *Grimontviridae* at the family level, together with genus- and species-level taxa such as *Obolenskvirus*, *Crifsvirus*, *Lactococcus phage Tuc2009* and *Clostridium phage vB_CpeS_CP51*.

To further explore potential ecological relationships between the gut virome and bacteriome during inflammatory arthritis, we performed a cross-domain Spearman correlation analysis between differentially abundant viral OTUs and bacterial genera identified in the control and model groups. Several significant virus–bacteria associations were identified, suggesting potential interaction patterns between components of the gut virome and bacterial community (Supplementary Fig. 1).

Together, these results suggested that K/BxN serum-transfer arthritis is accompanied by coordinated alterations of bacteriophage communities and their ecological associations with bacterial taxa, reflecting broader restructuring of the gut virome under inflammatory conditions.

### Functional prediction of the gut virome in K/BxN serum-transfer arthritis

**Fig. 3:**
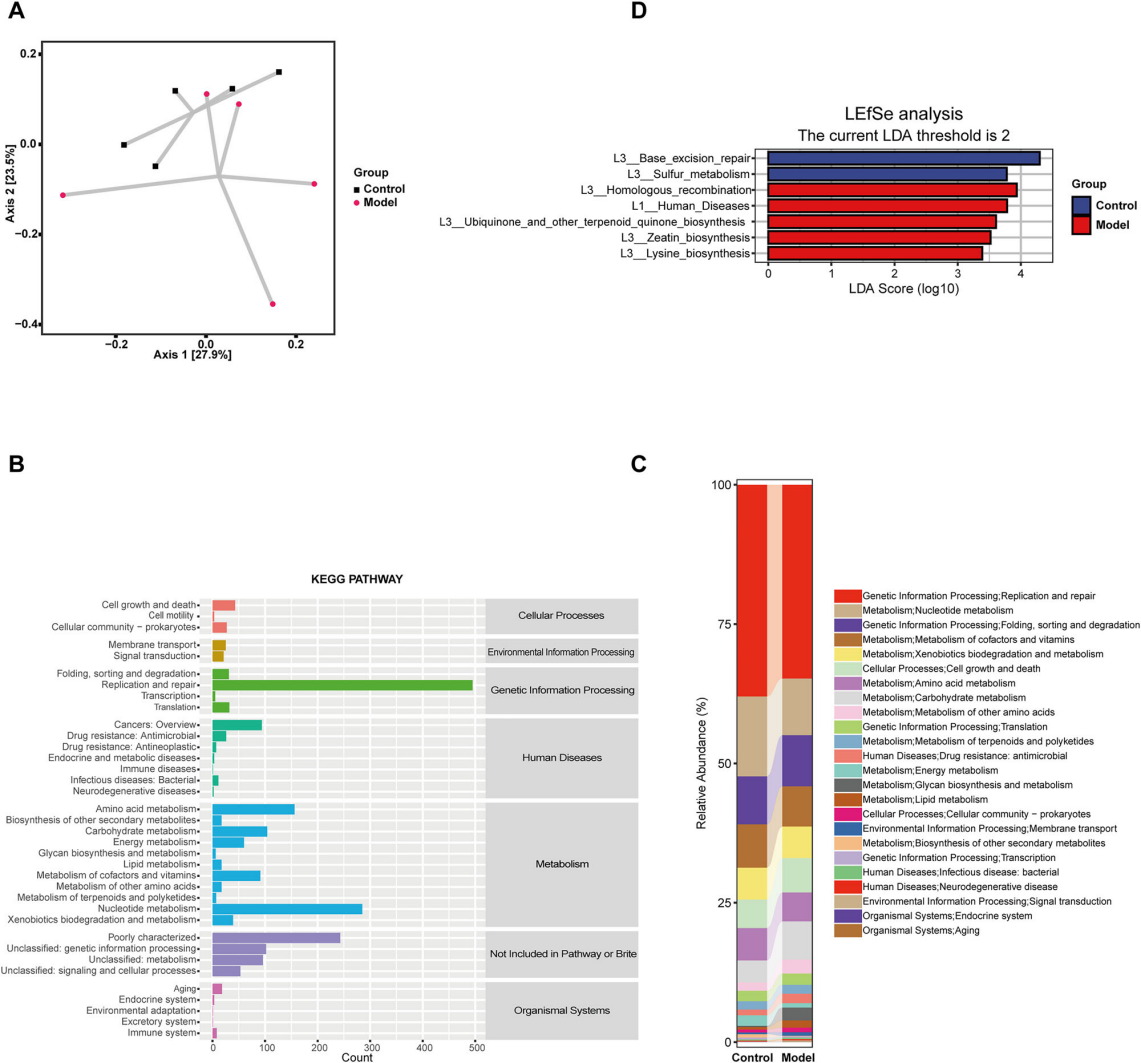
Functional prediction of gut virome in K/BxN serum-transfer arthritis mice. **A** PCoA of predicted viral functional profiles showing separation between groups. **B** KEGG classification of predicted viral genes. **C** Relative abundance of major functional categories. **D** LEfSe (LDA > 2.0) highlighting distinct functional pathways enriched in control and model groups.

To evaluate functional alterations of the gut virome, predicted viral genes were annotated against the KEGG database. PCoA based on functional profiles revealed a clear separation between control and model groups, suggesting differences in predicted functional repertoires (Fig. 3A). The majority of annotated functions were related to metabolism, genetic information processing, and cellular processes (Fig. 3B). Relative abundance analysis further showed that replication and repair, nucleotide metabolism, carbohydrate metabolism, and amino acid metabolism were the dominant categories across groups (Fig. 3C).

Functional enrichment analysis based on LEfSe (LDA > 2.0) showed that control mice were enriched in DNA repair and sulfur metabolism pathways, whereas model mice exhibited higher abundances of functions related to homologous recombination, disease-related pathways, and specialized biosynthetic pathways including ubiquinone, zeatin, and lysine biosynthesis (Fig. 3D).

To complement the KEGG-based analysis, additional functional annotations were performed using the GO, EggNOG, and CAZy databases. GO annotation confirmed that viral genes were predominantly enriched in cellular component and catalytic activity categories (Supplementary Fig. 2A). EggNOG classification further revealed replication/recombination/repair and transcription as the dominant functional categories. Consistently, LEfSe analysis (LDA > 2.0) indicated enrichment of control samples in signal transduction mechanisms, whereas model mice were enriched in carbohydrate transport and metabolism (Supplementary Fig. 2B, C). CAZy annotation identified glycoside hydrolases (GH) as the predominant enzyme family, followed by carbohydrate-binding modules (CBM). In agreement, LEfSe analysis (LDA > 2.0) showed enrichment of GH23 and CBM35 in the control group, while GT32 and CE9 modules were more abundant in the model group (Supplementary Fig. 2D, E).

Taken together, these results suggested that arthritis is characterized by functional remodeling of the gut virome, involving shifts in viral replication, repair, metabolic pathways, and carbohydrate-active enzyme modules.

### Preparation and characterization of fecal virus-like particles

**Fig. 4:**
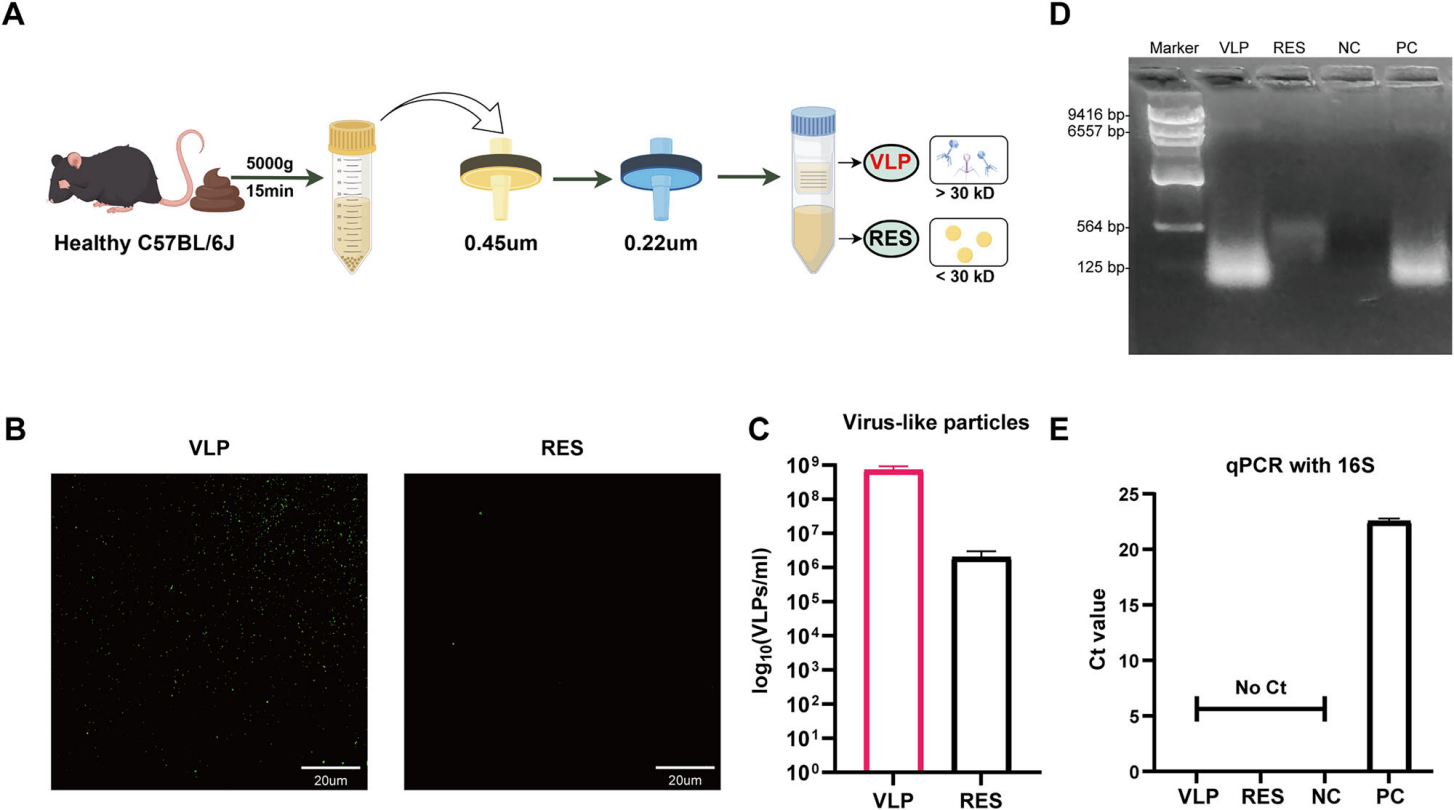
Preparation and characterization of fecal VLPs. **A** Workflow for isolating VLPs and RES fractions. **B** SYBR Gold staining of viral particles. **C** Quantification of viral particles in VLPs and RES fractions. **D** Agarose gel electrophoresis showing viral DNA without detectable bacterial genomic contamination. VLP virus-like particle fraction, RES residual fraction, NC blank extraction, PC total fecal viral nucleic acids before fractionation. **E** 16S rDNA qPCR confirming absence of bacterial DNA in VLPs and RES fractions. VLP virus-like particle fraction, RES residual fraction, NC no-template control, PC bacterial genomic DNA positive control.

The compositional and functional remodeling of the gut virome observed in arthritic mice suggests that viral components may be linked to disease-related processes. Based on this, we hypothesized that VLPs derived from healthy donors could serve as a potential therapeutic intervention. To test this, we established a standardized workflow for isolating viral particles from fecal samples of healthy C57BL/6 J mice, involving sequential centrifugation to remove debris, filtration through 0.45 μm and 0.22 μm membranes to remove cells and bacterial contaminants, and ultrafiltration with a 30 kDa cutoff to enrich VLPs while collecting virus-depleted RES (Fig. 4A). SYBR Gold staining revealed abundant fluorescent viral particles in the VLP fraction, whereas only sparse signals were detected in RES (Fig. 4B). Quantification confirmed that the VLP fraction contained more than 10⁸ particles/mL, markedly higher than RES (Fig. 4C). Agarose gel electrophoresis showed that nucleic acids extracted from VLPs appeared as diffuse bands ranging from approximately 100 bp to 9.4 kb, indicating a broad size distribution consistent with fragmented viral genomes commonly observed in fecal virome preparations. The positive control (total viral nucleic acids before fractionation) exhibited a similar broad pattern, suggesting that most viral genetic material was retained in the VLP fraction. In contrast, the RES fraction contained only faint low–molecular-weight fragments, and no bacterial genomic DNA was detected in any sample (Fig. 4D). Furthermore, 16S rDNA qPCR analysis confirmed the absence of bacterial contamination: both VLP and RES fractions showed undetectable Ct values, in contrast to the strong amplification observed in the positive bacterial control (Fig. 4E). Collectively, these results confirmed the successful preparation of concentrated viral particles, providing a standardized source for subsequent transplantation experiments.

### Attenuation of joint inflammation, systemic cytokines, and bone destruction following fecal virome transplantation

**Fig. 5:**
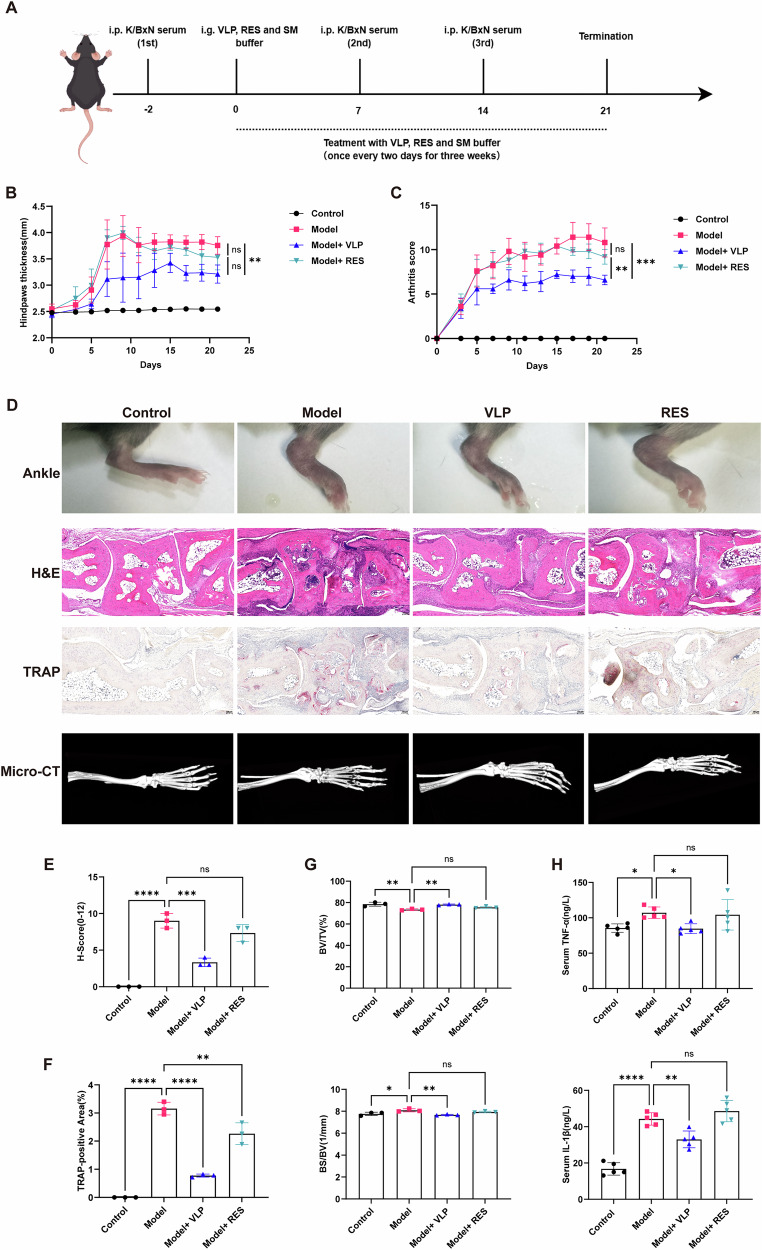
Mitigation of arthritis severity and bone destruction by fecal virome transplantation in K/BxN serum-transfer mice. **A** Experimental timeline. **B**, **C** Longitudinal analysis of hind paw thickness and arthritis scores. **D** Representative ankle images, H&E staining, TRAP staining, and micro-CT scans. **E** Quantification of synovial inflammation (H-score). **F** Percentage of TRAP-positive area. **G** Bone microstructural parameters (BV/TV, BS/BV) from micro-CT analysis. **H** Serum TNF-α and IL-1β concentrations. Data are presented as mean ± SD with individual values. *p < 0.05, **p < 0.01, ***p < 0.001, ****p < 0.0001; ns not significant.

To evaluate the therapeutic effects of fecal virome transplantation, K/BxN serum-transfer arthritis mice received VLPs, RES, or SM buffer by oral gavage every other day for three weeks (Fig. 5A). Compared with untreated arthritic mice, VLP treatment was associated with reduced paw swelling and lower arthritis scores throughout the disease course (Fig. 5B, C). Gross examination confirmed that ankle swelling was visibly alleviated in VLP-treated mice, whereas RES treatment exerted minimal improvement (Fig. 5D, upper panel).

Histopathological analysis revealed pronounced synovial hyperplasia, inflammatory infiltration, and cartilage erosion in the model group, all of which were significantly attenuated following VLPs administration (Fig. 5D, middle panel; Fig. 5E). TRAP staining demonstrated that VLPs significantly reduced osteoclast accumulation, indicating suppression of bone resorption (Fig. 5D, lower middle panel; Fig. 5F). Consistent with these findings, micro-CT imaging showed preservation of bone integrity in VLP-treated mice, whereas RES-treated mice remained comparable to the model group (Fig. 5D, bottom panel; Fig. 5G).

Moreover, serum cytokine analysis revealed that VLPs significantly reduced elevated IL-1β and TNF-α levels in arthritic mice, whereas RES treatment failed to improve systemic inflammation (Fig. 5H).

Overall, these results indicated that fecal virome transplantation via VLPs correlates with reduced joint inflammation, lower systemic cytokine levels, and improved bone structural integrity in K/BxN serum-transfer arthritis, whereas the virus-depleted RES fraction did not produce comparable effects.

### Alteration of myeloid responses in the synovium, spleen, and peripheral blood following fecal virome transplantation

**Fig. 6:**
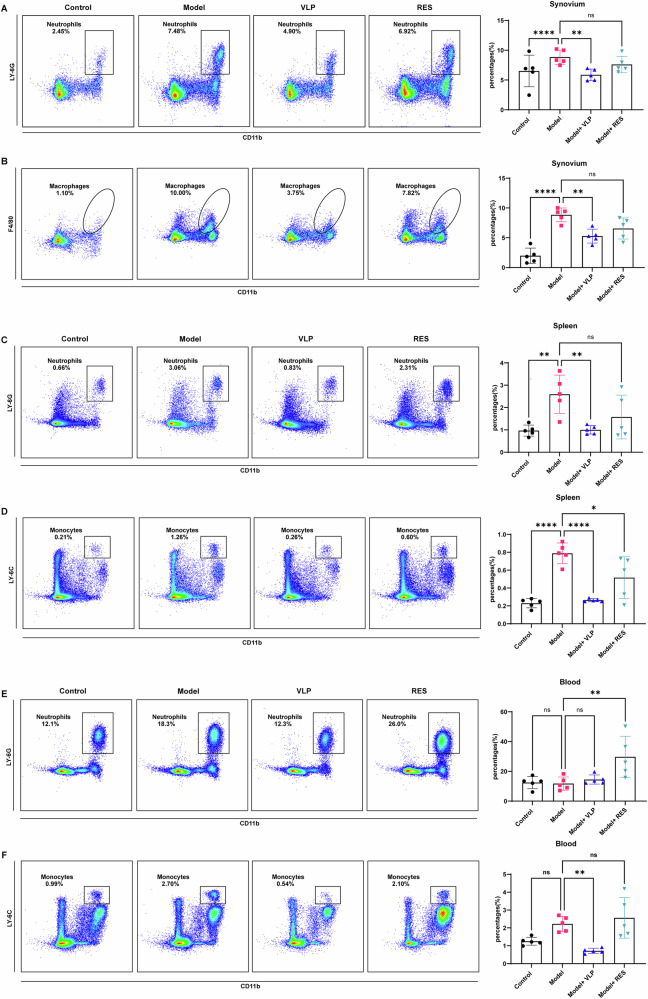
Flow cytometric analysis of myeloid cell subsets in arthritic mice. **A**, **B** Neutrophils (Ly6G⁺CD11b⁺) and macrophages (F4/80⁺CD11b⁺) in the synovium. **C**, **D** Neutrophils and monocytes (Ly6C⁺CD11b⁺) in the spleen. **E**, **F** Neutrophils and monocytes in peripheral blood. Data are presented as mean ± SD with individual values. Statistical analysis was performed using one-way ANOVA followed by Dunnett’s multiple comparisons test, with the model group set as reference. *p < 0.05, **p < 0.01, ***p < 0.001, ****p < 0.0001, ns not significant.

To further explore the immunological changes associated with VLP administration, we performed flow cytometric profiling of myeloid populations across multiple compartments (Fig. 6).

In the synovium, both neutrophils and macrophages were markedly increased in arthritic mice, whereas VLP treatment corresponded to reduced infiltration of these inflammatory cells (Fig. 6A, B). In contrast, RES showed no significant improvement relative to the model group.

In the spleen, neutrophils and monocytes were significantly elevated in arthritic mice. VLP administration was associated with reductions in both populations, restoring their levels toward those of healthy controls (Fig. 6C, D). While RES partially reduced splenic monocytes, its effect was still inferior to VLPs and insufficient to alleviate neutrophil expansion.

In peripheral blood, neutrophil proportions were comparable between the model and control groups, and VLP intervention had no significant effect on this systemic compartment. Interestingly, RES treatment was accompanied by an increase in circulating neutrophils, suggesting a potential role for components retained in RES in influencing systemic myeloid cell mobilization (Fig. 6E). Circulating monocytes showed modest variation, with VLPs tending to reduce their frequency, while RES exhibited no beneficial effects (Fig. 6F).

Collectively, these results indicated that while K/BxN arthritis involves prominent myeloid activation, VLPs primarily exert a localized anti-inflammatory effect in the affected joint and spleen, without inducing systemic immune suppression. Conversely, RES may be linked to increased peripheral myeloid activation, supporting the notion that the viral particle–enriched fraction exhibits distinct biological activity compared with the virus-depleted preparation.

### Remodeling of gut microbial composition and structure following fecal virome transplantation

**Fig. 7:**
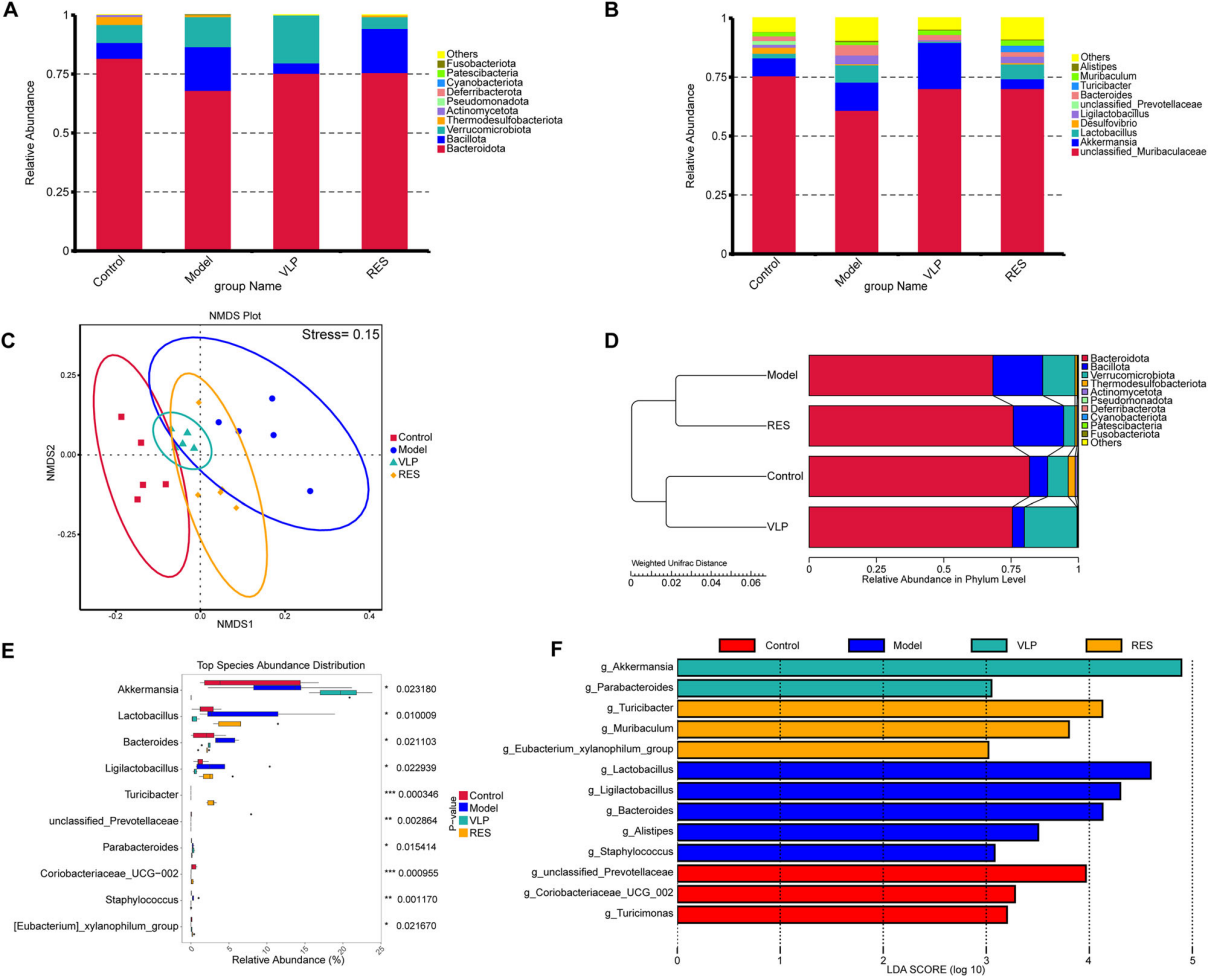
Remodeling of gut microbial composition and structure following fecal virome transplantation. **A** Relative abundance of dominant bacterial phyla and **B** genera. **C** NMDS analysis of β-diversity based on Bray–Curtis distance. **D** Hierarchical clustering using weighted UniFrac distance. **E** Kruskal–Wallis H test of significantly different genera among groups. **F** LEfSe analysis (LDA > 3.0) showing representative taxa enriched in each group. Control healthy control, Model arthritic mice, VLP virus-like particle-treated, RES filtrate-treated.

To explore changes in gut microbiota composition following FVT in arthritic mice, bacterial community structures were compared among groups. At the phylum level, *Bacteroidota* and *Firmicutes* dominated across all groups, with *Verrucomicrobiota* showing a modest increase in the VLP group (Fig. 7A). At the genus level, *Akkermansia*, *Lactobacillus*, and *Bacteroides* displayed notable variations, with *Akkermansia* being relatively enriched in the VLP group (Fig. 7B).

Non-metric multidimensional scaling (NMDS) analysis based on β-diversity revealed partial separation among groups. The VLP group clustered closer to the controls, whereas the RES group occupied an intermediate position (Fig. 7C). Hierarchical clustering analysis showed a similar trend, where the VLP group was positioned nearer to the control cluster, while the model and RES groups formed distinct branches (Fig. 7D).

Differential abundance analysis using the Kruskal–Wallis test identified several genera with significant differences among groups, including *Akkermansia*, *Lactobacillus*, and *Turicibacter* (Fig. 7E). Consistently, LEfSe analysis (LDA > 3.0) identified distinct bacterial biomarkers among groups. The VLP group was characterized by enrichment of *Akkermansia* and *Parabacteroides*, while the Model group showed dominance of *Lactobacillus* and *Bacteroides*. Additionally, *Turicibacter* was relatively enriched in the RES group, whereas *unclassified Prevotellaceae* appeared more abundant in the Control group (Fig. 7F).

To examine the relationship between *Akkermansia* abundance and disease severity, we performed Spearman correlation analysis in mice from the Model and VLP groups (*n* = 10). *Akkermansia* abundance showed a moderate negative trend with arthritis clinical scores (*r* = −0.57, *p* = 0.09), although this did not reach statistical significance (Supplementary Fig. 3A). In addition, *Akkermansia*-centered co-occurrence networks were constructed for the control, model, and VLP groups. The resulting association patterns differed markedly among groups (Supplementary Fig. 3B).

Taken together, these findings suggested that FVT, particularly VLP treatment, coincided with shifts in the gut microbial community toward a composition more similar to that of healthy controls, suggesting a partial restoration of microbial homeostasis.

### Reshaping of microbial community assembly and systemic tryptophan metabolism following fecal virome transplantation

To explore ecological processes associated with FVT intervention, we first examined microbial community assembly and predicted functional profiles. The Model group exhibited broader βNTI distributions and higher NST values, indicating greater stochastic influence in microbial community assembly. In contrast, the VLP group showed reduced NST values and narrower βNTI distributions, suggesting a shift toward more deterministic assembly patterns (Supplementary Fig. 4A, B). Functional prediction based on PICRUSt2 further indicated global metabolic disturbance in the arthritic state. Notably, pathways related to amino acid metabolism—including phenylalanine, tyrosine, and tryptophan biosynthesis—were relatively enriched following VLP treatment (Supplementary Fig. 4C–E), suggesting potential alterations in microbial tryptophan metabolism.

**Fig. 8:**
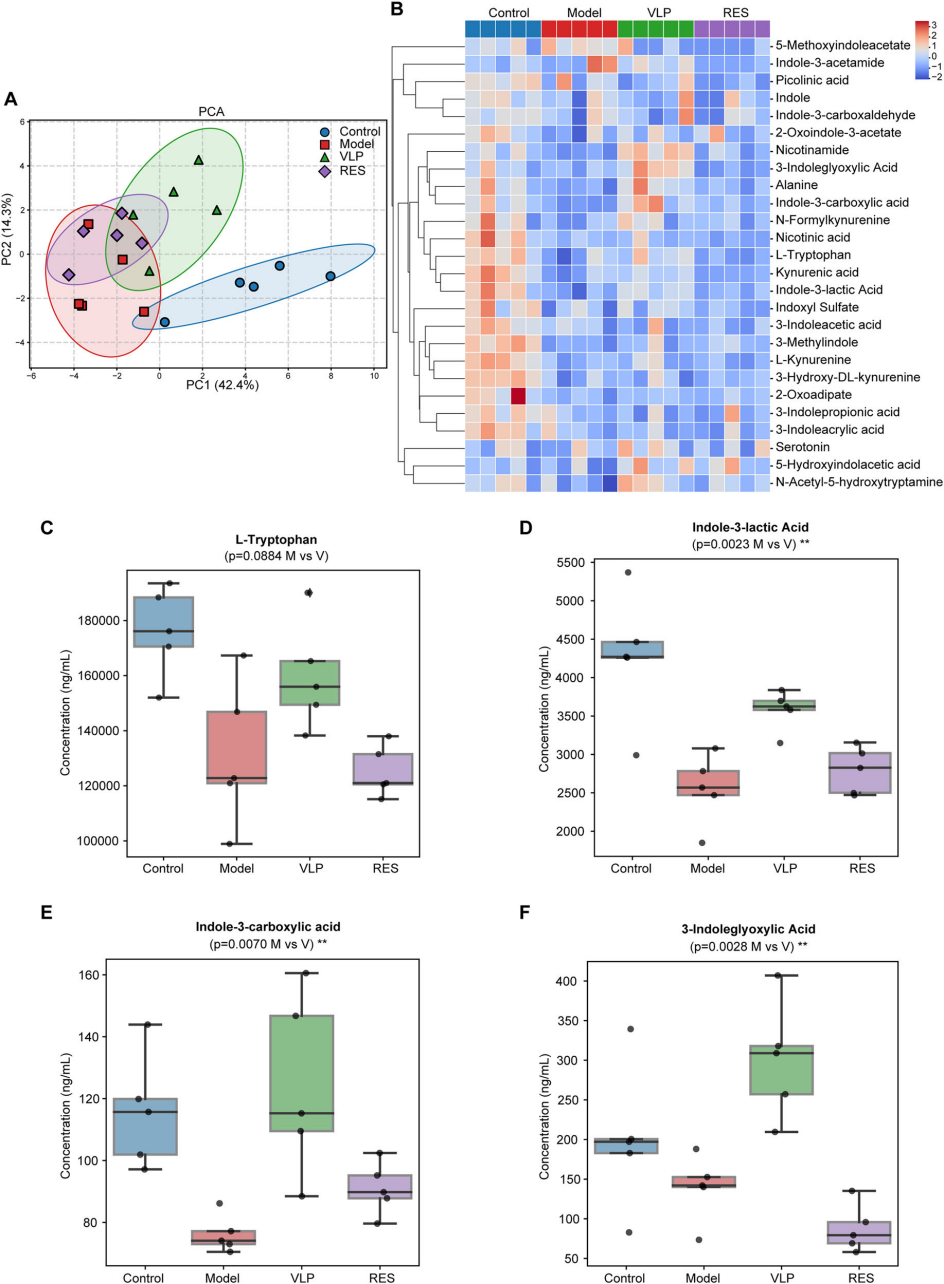
Targeted metabolomic analysis of tryptophan-derived metabolites following fecal virome transplantation. **A** Principal component analysis (PCA) of targeted tryptophan metabolites showing separation among Control, Model, VLP, and RES groups. **B** Heatmap illustrating relative abundance of tryptophan-related metabolites across samples. **C** Relative abundance of L-tryptophan among the four groups. **D** Relative abundance of indole-3-lactic acid. **E** Relative abundance of indole-3-carboxylic acid. **F** Relative abundance of 3-indoleglyoxylic acid. Data are presented as boxplots with individual data points. Statistical significance was evaluated using appropriate group comparisons. **p < 0.01, ns not significant.

Because microbiota-derived tryptophan metabolites could enter the host circulation and influence systemic immune responses, we next examined whether these predicted microbial functional changes were reflected in host metabolic profiles. Targeted metabolomic analysis of tryptophan-related metabolites was therefore performed using serum samples. Multivariate analysis revealed clear separation among the four groups, indicating distinct metabolic profiles (Fig. 8A). Notably, the metabolic profile of the VLP group remained clearly separated from the untreated Model group, whereas the RES group clustered closely with the Model group. Consistent with this observation, hierarchical clustering heatmap analysis revealed marked differences in tryptophan-derived metabolites across groups, with several indole metabolites showing partial restoration following VLP treatment (Fig. 8B). At the individual metabolite level, circulating L-tryptophan levels showed a decreasing trend in arthritic mice and a partial recovery following VLP treatment (Fig. 8C). More importantly, several microbiota-derived indole metabolites were significantly increased in the VLP group compared with the Model group, including indole-3-lactic acid (ILA) (Fig. 8D), indole-3-carboxylic acid (Figs. 8E), and 3-indoleglyoxylic acid (Fig. 8F). In contrast, administration of the virus-depleted residual fraction (RES) did not restore these metabolites.

Overall, these findings suggested that FVT is associated with alterations in microbial community assembly and enhanced production of microbiota-derived tryptophan metabolites, particularly indole derivatives that have been implicated in host immune regulation.

## Discussion

Rheumatoid arthritis (RA) is characterized by chronic joint inflammation driven by dysregulated immune responses. Increasing evidence suggests that the gut microbiome contributes to systemic immune modulation and disease progression^22^. While most previous studies have focused on bacterial dysbiosis, the potential role of the gut virome remains largely unexplored^8^. In the present study, we provide experimental evidence that fecal virome transplantation (FVT) from healthy donors correlates with attenuation of inflammatory responses in a serum-induced arthritis model. VLP administration was linked to reduced paw swelling, decreased systemic cytokine levels, and preservation of bone structure, accompanied by alterations in gut microbial composition and metabolic profiles.

The restructuring of the gut virome observed in arthritic mice provides ecological context for these findings. For instance, enrichment of phage taxa such as *Paclarkvirus* and *Thalassavirus* in the arthritis model suggests altered bacteriophage–bacterium dynamics under inflammatory conditions. According to RefSeq annotations, *Paclarkvirus* phages have been reported to infect *Streptococcus* species^23^, whereas *Thalassavirus* phages have been associated with *Vibrio* hosts^24^. Although host specificity within the murine gut cannot be directly inferred from sequence-based classification alone, the differential abundance of these phage taxa likely reflects shifts in cross-kingdom ecological interactions within the gut ecosystem. Importantly, the increased virome diversity observed in arthritic mice should not necessarily be interpreted as intrinsically pathogenic or disease-promoting. Instead, such changes may reflect ecological instability or secondary responses to bacterial dysbiosis and inflammatory stress.

Consistent with the clinical improvements observed following VLP administration, flow cytometric analysis revealed reductions in neutrophil and monocyte/macrophage populations in the synovium and spleen. These observations suggest that VLP treatment was associated with modulation of innate inflammatory effector responses rather than generalized systemic immunosuppression. In contrast, administration of the virus-depleted residual fraction (RES) did not provide comparable protection and was accompanied by increased circulating neutrophils, indicating that components retained in the viral particle–enriched fraction may contribute to the observed immunological changes.

FVT also coincided with shifts in the bacterial microbiota, including enrichment of *Akkermansia*. Previous studies have reported that *Akkermansia* can exhibit context-dependent effects, being associated with either inflammatory activation or mucosal protection depending on host physiological conditions^25–28^. For example, dietary factors such as high-fiber intake may increase *Akkermansiaceae* abundance and interact with taxa such as *Prevotella copri* to influence inflammatory responses^26^. In contrast, enrichment of *Akkermansia* together with beneficial taxa such as *Bifidobacterium* has been correlated with improved arthritis outcomes^28^. Previous studies have reported both beneficial and detrimental associations of *Akkermansia* in inflammatory diseases, suggesting that its role may depend on the surrounding microbial ecosystem. Our findings support this ecological perspective, indicating that *Akkermansia* enrichment may reflect broader restructuring of the gut microbial community rather than acting as an isolated driver of disease modulation.

In our serum-induced arthritis model, VLP administration was accompanied by attenuation of inflammatory responses, accompanied by changes in both the gut virome and bacteriome. These observations suggest that modulation of the gut virome may influence host inflammation through alterations in cross-kingdom ecological interactions within the gut ecosystem. Consistent with this interpretation, cross-domain association patterns between viral and bacterial taxa were observed, indicating coordinated restructuring of selected components of the gut microbial community during inflammatory arthritis. However, correlation analyses cannot directly distinguish specific phage–host interactions from broader ecological co-variation within the microbial ecosystem.

Besides taxonomic alterations, functional analyses suggested that FVT was linked to changes in microbial metabolic potential, particularly pathways related to amino acid metabolism. Amino acid metabolism represents a critical component of immunometabolism and plays an important role in modulating host inflammatory responses and immune homeostasis in inflammatory diseases^29^. Among these pathways, tryptophan metabolism represents a key interface linking the gut microbiota and host immune regulation. Disturbances in the tryptophan–indole metabolic axis have been implicated in inflammatory disorders including arthritis^30,31^. Microbiota-derived tryptophan metabolites could cross the intestinal barrier and enter the host circulation, thereby linking gut microbial activity to systemic immunometabolic regulation^6^. Consistent with this concept, targeted metabolomic analysis in the present study revealed increased levels of several indole metabolites following VLP treatment, including indole-3-lactic acid (ILA), indole-3-carboxylic acid, and 3-indoleglyoxylic acid. These indole derivatives have been reported to interact with host immune pathways such as aryl hydrocarbon receptor (AhR) signaling, which plays an important role in maintaining mucosal immune homeostasis^32,33^. Therefore, the observed metabolic changes may represent a potential link between virome-associated ecological remodeling and host immunometabolic regulation.

From a conceptual perspective, our findings suggest that the gut virome may represent a previously underappreciated component of the gut–joint axis. Compared with fecal microbiota transplantation (FMT), FVT selectively transfers viral particles while excluding live bacteria, which may theoretically reduce the risk of pathogen transmission and uncontrolled bacterial colonization^34–36^. By influencing microbial ecological networks rather than directly introducing new bacterial communities, FVT provides a valuable experimental framework for investigating virus–bacteria interactions under inflammatory conditions, including processes relevant to inflammatory arthritis. The potential biological significance of these interactions warrants further investigation in future studies.

Several limitations should be acknowledged. First, the K/BxN serum-transfer model primarily represents the antibody-driven effector phase of arthritis, which does not fully recapitulate the adaptive immune priming characteristic of human RA^21^. Future studies using adaptive immune-dependent models, such as collagen-induced arthritis (CIA), are needed. Second, while VLP administration was associated with arthritis attenuation and gut microbiota remodeling, direct viral causality cannot be definitively established, and the observed benefits are likely mediated through virus–bacteria ecological interactions. Finally, specific phage–host relationships and viral engraftment dynamics were not experimentally tracked. Future investigations integrating strain-resolved metagenomics and phage isolation will be crucial to decipher these cross-domain interactions within the gut ecosystem.

Taken together, these findings indicated that fecal virome transplantation was associated with attenuation of serum-induced arthritis, accompanied by alterations in gut microbial ecology and tryptophan-related metabolic profiles. These findings highlight the gut virome as a previously underappreciated component of the gut–joint axis and provide a framework for future studies investigating the role of the gut virome in inflammatory arthritis.

## Methods

### Animals and experimental design

Six- to eight-week-old female C57BL/6 J mice were purchased from Guangdong GemPharmatech Co., Ltd., China and maintained under specific pathogen-free (SPF) conditions (22 ± 2 °C, 55 ± 5% humidity, 12-h light/dark cycle), with ad libitum access to sterile food and water. All mice were kept in the Laboratory Animal Center of Jinan University (SPF environment) and acclimated for at least one week before experiments. All animal experiments were performed in accordance with the rules and ethics for animal experiments of the Animal Experimental Center, Jinan University, Guangzhou, China (IACUC No. 20241119-06).

The K/BxN serum-transfer arthritis model was induced by intraperitoneal injection of 100 μL arthritogenic K/BxN serum on days 0, 7, and 14. Mice were randomly assigned to four groups: Control, Model, Model + VLP, and Model + RES. Viral-like particles (VLPs; 10⁸ particles/mL, 200 μL per mouse) or the corresponding virus-depleted filtrate residual fluid (RES) were administered via oral gavage, using SM buffer (NaCl 200 mM, MgSO₄·7H₂O 16 mM, Tris–HCl 100 mM, pH 7.5) as the vehicle control. All mice were pretreated with 50 μL of 1 M NaHCO₃ solution prior to each gavage to neutralize gastric acid and enhance viral particle survival. Treatments were performed every other day for three consecutive weeks. Fresh fecal samples were collected weekly to prepare viral materials for continuous intervention. Paw swelling and clinical arthritis scores were recorded every two days throughout the experiment.

### Histopathological Evaluation (H&E Staining)

Fresh ankle joints were harvested after euthanasia and fixed in 4% paraformaldehyde for 48 h (tissue: fixative > 1:20). After trimming, PBS rinsing, dehydration, clearing, paraffin embedding, and sectioning at 5 μm thickness, the samples were stained with hematoxylin and eosin (H&E). Histopathological evaluation was performed by blinded investigators using a semi-quantitative scoring system, in which synovial hyperplasia, inflammatory cell infiltration, and cartilage/bone destruction were graded from 0 to 4 and summed to yield a total score of 0–12 to assess arthritis severity.

### Tartrate-resistant acid phosphatase (TRAP) staining

Fresh ankle joints were collected after euthanasia and fixed in 4% paraformaldehyde for 48 h (tissue: fixative ratio > 1:20) to preserve tissue integrity. After fixation, specimens were dehydrated, cleared in xylene, embedded in paraffin, and sectioned at 5 μm thickness. Sections were baked, deparaffinized, rehydrated, and subjected to TRAP staining. TRAP-positive osteoclasts were identified as multinucleated cells with ≥3 nuclei showing dark red or purple precipitates. Five randomly selected high-power fields (HPF) per section were imaged, and quantitative analysis of TRAP-positive cell numbers and the percentage of TRAP-positive area was performed using ImageJ software to evaluate osteoclast activity and bone resorption.

### Micro-CT scanning and analysis

Micro-CT imaging of the ankle joints was performed using an InSyTe small animal tomography system (TriFoil Imaging, USA). The scanning parameters were set at 40 kV and 750 μA, with an isotropic voxel size of 37.5 μm. Projection images were reconstructed and visualized using vivoQuant software (inviCRO, USA). Quantitative bone morphometric analyses, including BV/TV and BS/BV, were carried out using MicroView software (GE Healthcare, USA) to evaluate the severity of bone erosion.

### Enzyme-Linked Immunosorbent Assay (ELISA)

Peripheral blood was collected from mice, and samples were centrifuged at 3000 rpm for 15 min to obtain serum, which was stored at −80 °C until analysis. Serum levels of inflammatory cytokines (e.g., TNF-α, IL-1β) were measured using commercial ELISA kits (MEIMIAN, Jiangsu, China). Serially diluted standards were used to generate standard curves, and absorbance was read at 450 nm. All samples were assayed in duplicate, and cytokine concentrations were calculated based on the standard curves and expressed as mean ± SD.

### Targeted metabolomic analysis of tryptophan metabolites

Serum samples were subjected to targeted metabolomic analysis of tryptophan-related metabolites using ultra-high performance liquid chromatography–tandem mass spectrometry (UHPLC–MS/MS). Briefly, serum samples were diluted with ultrapure water and vortex-mixed. A 100 μL aliquot of the diluted sample was mixed with 300 μL of 80% methanol containing mixed internal standards, vortexed thoroughly, and incubated on ice for 10 min to precipitate proteins. Samples were centrifuged at 15,000 rpm for 15 min, and the supernatant was collected for LC–MS/MS analysis. Metabolite detection was performed using an ExionLC™ AD UHPLC system coupled with a QTRAP® 6500+ mass spectrometer (AB SCIEX, USA). Quantification was conducted in multiple reaction monitoring (MRM) mode using authentic standards.

### Metavirome sequencing

Fecal samples were processed by differential centrifugation and filtration through a 0.22-μm membrane to remove host cells and bacteria. Virus-like particles were enriched using PEG6000/NaCl precipitation and treated with DNase to eliminate free nucleic acids. Viral DNA/RNA was extracted using commercial kits, followed by library preparation with the Illumina TruSeq DNA protocol or the NEBNext Ultra II RNA Library Prep Kit. Sequencing was performed on an Illumina NovaSeq platform with paired-end reads. Quality control, assembly, taxonomic classification, and functional annotation were performed using fastp, metaSPAdes, and multiple databases including KEGG, EggNOG, GO, and CAZy, enabling comparative analysis of viral community structure and predicted functional pathways. Taxonomic annotation of viral contigs was performed using DIAMOND against the NCBI RefSeq viral database. To explore potential ecological relationships between the gut virome and bacteriome, cross-domain Spearman correlation analysis was performed between differentially abundant viral OTUs and bacterial genera using matched metavirome and 16S datasets derived from the same fecal samples. Correlations with |r | ≥ 0.6 and *p* < 0.05 were retained for visualization.

### 16S rRNA gene sequencing

Total microbial DNA was extracted from fecal samples using a commercial extraction kit. The V3–V4 region of the bacterial 16S rRNA gene was amplified with universal primers 338 F/806 R. PCR products were purified, quantified, pooled equimolarly, and used for library construction with the Illumina TruSeq Nano DNA LT Library Prep Kit. Sequencing was conducted on the NovaSeq6000 platform (paired-end mode). Bioinformatic analysis was performed using QIIME2 for quality filtering, ASV generation, and taxonomic assignment, followed by α- and β-diversity assessment and differential abundance analysis (LEfSe, PERMANOVA) to characterize alterations in gut bacterial composition among experimental groups. The same fecal samples used for metavirome sequencing were also subjected to 16S rRNA gene sequencing to enable cross-domain analyses between viral and bacterial communities. To further investigate microbial associations related to *Akkermansia*, co-occurrence network analysis was performed separately for the control, model, and VLP groups. Spearman correlation matrices were calculated using differentially abundant bacterial taxa. Correlations with |r | ≥ 0.6 and *P* < 0.05 were retained, self-correlations were removed, and taxa with relative abundance <0.005% were excluded.

### Isolation, verification, and quality control of VLPs and RES fractions

Fresh fecal pellets from 10 healthy female C57BL/6 J donor mice (6–8 weeks old) were collected for VLP isolation. Donor mice were age- and sex-matched, maintained under SPF conditions, and free of clinical abnormalities. Fecal samples from all donors were pooled prior to VLP isolation to minimize individual variability and generate a representative virome preparation. The pooled samples were homogenized on ice and centrifuged at 5000 × *g* for 15 min at 4 °C to remove large debris. After sequential filtration through 0.45 μm and 0.22 μm membranes to remove cells and bacteria, the filtrate was further processed by 30 kDa centrifugal ultrafiltration (4000 × *g*, 10 min, 4 °C). The retentate (>30 kDa), enriched in virus-like particles, was collected as the VLP fraction, whereas the filtrate (<30 kDa), depleted of viral components, was designated as the RES fraction.

Viral nucleic acids were extracted from both VLPs and RES samples using the HiPure Viral RNA/DNA Kit (Magen Biotechnology, China, Cat# R4173-02). Nucleic acid concentration and purity were assessed by NanoDrop, and 1% agarose gel electrophoresis was performed to examine nucleic acid integrity and exclude high-molecular-weight bacterial genomic DNA. Bacterial contamination was further checked via 16S rRNA gene qPCR using universal primers (27 F/1492 R), and samples with Ct > 35 or no amplification were considered free of bacterial residues.

VLPs were visualized by epifluorescence microscopy following SYBR Gold staining (10000×, LABLEAD Biotech, China, Cat# CG002), as described in the protocol available at: 10.17504/protocols.io.bx6cpraw. Only samples that passed all QC procedures were utilized for oral gavage administration in mice.

### Flow cytometry analysis

Single-cell suspensions were prepared from the ankle joints, spleens, and peripheral blood of mice. Ankle joints were minced and digested in RPMI-1640 medium containing 2% FBS, collagenase D (50 μg/mL), and DNase I (20 μg/mL) at 37 °C with shaking (225 rpm) for 45 min, followed by filtration through a 70 μm cell strainer and centrifugation at 400 × *g* for 10 min. The cells were then resuspended in 35% Percoll and centrifuged at 600 × *g* for 25 min (acceleration 6, deceleration 2) for density-gradient separation. Spleens were mechanically dissociated, filtered through a 40 μm strainer, centrifuged at 400 × *g* for 5 min at 4 °C, treated with RBC lysis buffer, and washed with PBS. Peripheral blood was collected into EDTA-coated tubes and treated with 3–5 volumes of RBC lysis buffer for 10 min at room temperature, then washed twice and resuspended in PBS.

Cells were stained using a Zombie NIR Fixable Viability Kit (BioLegend, USA) to exclude dead cells, followed by surface staining with fluorochrome-conjugated antibodies at 4 °C for 30 min in the dark. After PBS washing, data were acquired using a Cytek flow cytometer (Cytek Biosciences, USA). The following antibodies were used (all from BioLegend, USA): BV421 anti-mouse CD11c, Zombie NIR Fixable Viability Kit, BV605 anti-mouse Ly6G, BV711 anti-mouse F4/80, FITC anti-mouse CD45, PerCP-Cy5.5 anti-mouse Ly6C, APC anti-mouse CD11b, and Alexa Fluor 647 anti-mouse MHC II.

For each sample, at least 10,000 CD45⁺ events were acquired and analyzed using FlowJo software (Tree Star, USA). Gating strategy: debris exclusion → singlets → viable cells → CD45⁺ leukocytes → myeloid subsets. All analyses were performed in a blinded manner, and data are presented as percentages.

### Statistical analysis

All data are presented as mean ± SD. Statistical analyses were performed using GraphPad Prism (Version 10.1.2, GraphPad Software Inc., USA). For analyses involving only two groups, unpaired two-tailed Student’s *t* tests were performed. For comparisons among multiple groups, one-way ANOVA followed by Dunnett’s multiple comparisons test was used, with the model group set as the reference to assess treatment effects. For β-diversity comparisons of microbial communities, permutational multivariate analysis of variance (PERMANOVA) based on Bray–Curtis distance with 999 permutations was performed using the vegan package in R. *P* < 0.05 was considered statistically significant.

## Supplementary information

41522_2026_980_MOESM1_ESM

41522_2026_980_MOESM2_ESM

## Data Availability

The 16S rRNA sequencing data and metavirome sequencing data generated in this study have been deposited in the NCBI BioProject database under accession numbers PRJNA1435886 and PRJNA1436163, respectively. All other data supporting the findings of this study are available from the corresponding author upon reasonable request.
