# Supplementary material for: Fecal virome transplantation attenuates arthritis in mice by remodeling gut ecology, systemic tryptophan metabolism, and innate immune responses
Source: NPJ Biofilms Microbiomes. 2026 Apr 8;12:111. doi: 10.1038/s41522-026-00980-2 (PMC13236970; doi:10.1038/s41522-026-00980-2)
Supplement: Supplementary file 1 — 41522_2026_980_MOESM1_ESM [file 41522_2026_980_MOESM1_ESM.pdf]

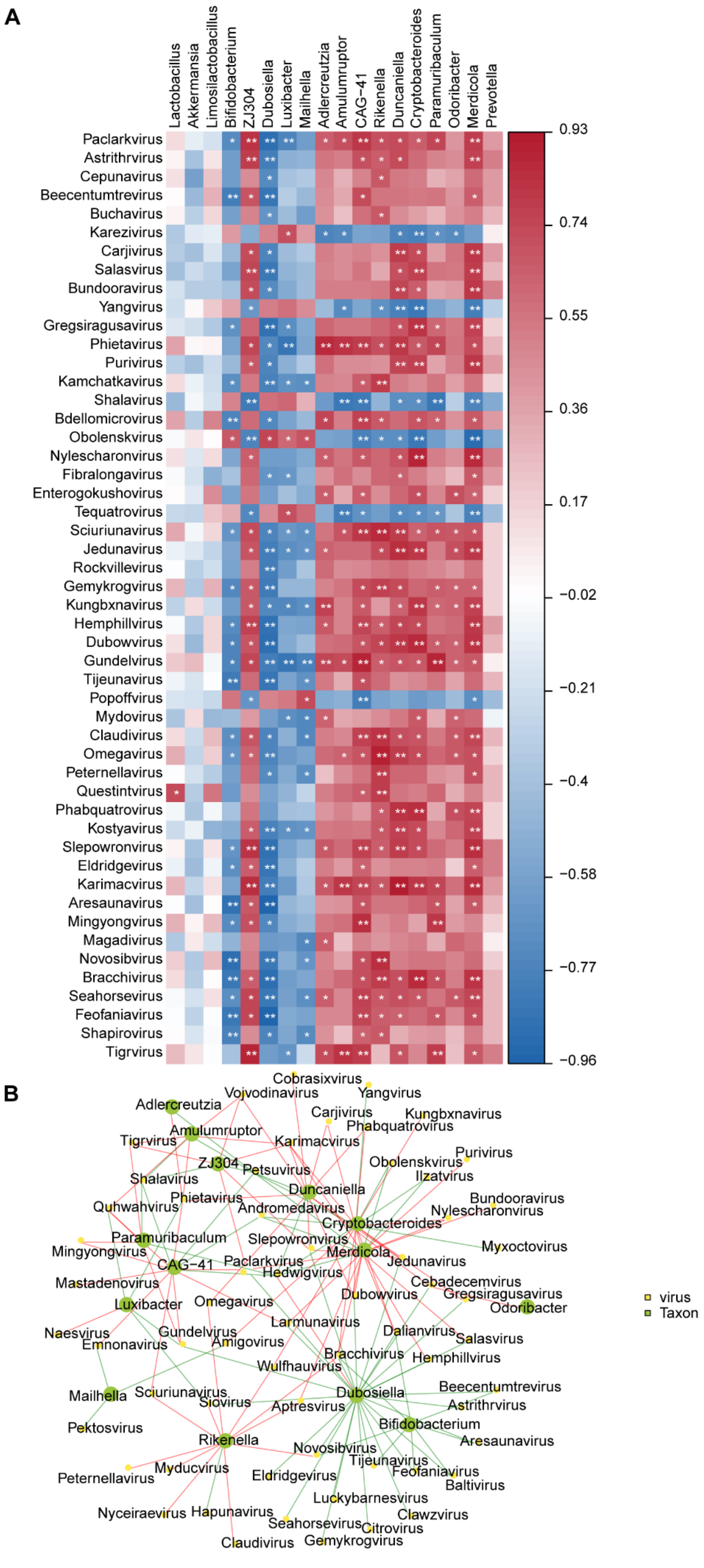
A
Lactobacillus
Akkermansia
Limosilactobacillus
Bifidobacterium
ZJ304
Dubosiella
Luxibacter
Mailhella
Adlercreutzia
Amulumruptor
CAG−41
Rikenella
Duncaniella
Cryptobacteroides
Paramuribaculum
Odoribacter
Merdicola
Prevotella
Paclarkvirus
Astrithrvirus
Cepunavirus
Beecentumtrevirus
Buchavirus
Karezivirus
Carjivirus
Salasvirus
Bundooravirus
Yangvirus
Gregsiragusavirus
Phietavirus
Purivirus
Kamchatkavirus
Shalavirus
Bdellomicrovirus
Obolenskvirus
Nylescharonvirus
Fibralongavirus
Enterogokushovirus
Tequatrovirus
Sciuriunavirus
Jedunavirus
Rockvillevirus
Gemykrogvirus
Kungbxnavirus
Hemphillvirus
Dubowvirus
Gundelvirus
Tijeunavirus
Popoffvirus
Mydovirus
Claudivirus
Omegavirus
Peternellavirus
Questintvirus
Phabquatrovirus
Kostyavirus
Slepowronvirus
Eldridgevirus
Karimacvirus
Aresaunavirus
Mingyongvirus
Magadivirus
Novosibvirus
Bracchivirus
Seahorsevirus
Feofaniavirus
Shapirovirus
Tigrvirus
0.93
0.74
0.55
0.36
0.17
−0.02
−0.21
−0.4
−0.58
−0.77
−0.96
B
Cobrasixvirus
Adlercreutzia
Vojvodinavirus
Yangvirus
Carjivirus
Kungbxnavirus
Phabquatrovirus
Amulumruptor
Tigrvirus
Karimacvirus
Purivirus
ZJ304
Petsuvirus
Obolenskvirus
Ilzatvirus
Shalavirus
Duncaniella
Phietavirus
Bundooravirus
Quhwahvirus
Andromedavirus
Nylescharonvirus
Cryptobacteroides
Paramuribaculum
Slepowronvirus
Myxoctovirus
Mingyongvirus
Merdicola
CAG−41
Paclarkvirus
Jedunavirus
Hedwigvirus
Cebadecemvirus
Mastadenovirus
Gregsiragusavirus
Dubowvirus
Luxibacter
Omegavirus
Odoribacter
Larmunavirus
Naesvirus
Gundelvirus
Dalianvirus
Salasvirus
Emnonavirus
Amigovirus
Bracchivirus
Hemphillvirus
Wulfhauvirus
Beecentumtrevirus
Mailhella
Sciuriunavirus
Dubosiella
Siovirus
Astrithrvirus
Aptresvirus
Bifidobacterium
Pektosvirus
Rikenella
Aresaunavirus
Novosibvirus
Tijeunavirus
Myducvirus
Feofaniavirus
Peternellavirus
Eldridgevirus
Baltivirus
Luckybarnesvirus
Nyceiraevirus
Hapunavirus
Clawzvirus
Seahorsevirus
Citrovirus
Claudivirus
Gemykrogvirus
virus
Taxon

Supplementary Figure 1. Cross-domain correlation analysis between differentially abundant viral and bacterial taxa in control and arthritic mice.

(A) Spearman correlation heatmap showing associations between differentially abundant viral genera and bacterial genera identified between the Control and Model groups. Red and blue colors indicate positive and negative correlations, respectively. Color intensity represents the Spearman correlation coefficient (r). *$p < 0.05$, **$p < 0.01$.

(B) Correlation network illustrating significant cross-domain associations between viral taxa (yellow nodes) and bacterial genera (green nodes). Red edges indicate positive correlations, and green edges indicate negative correlations.

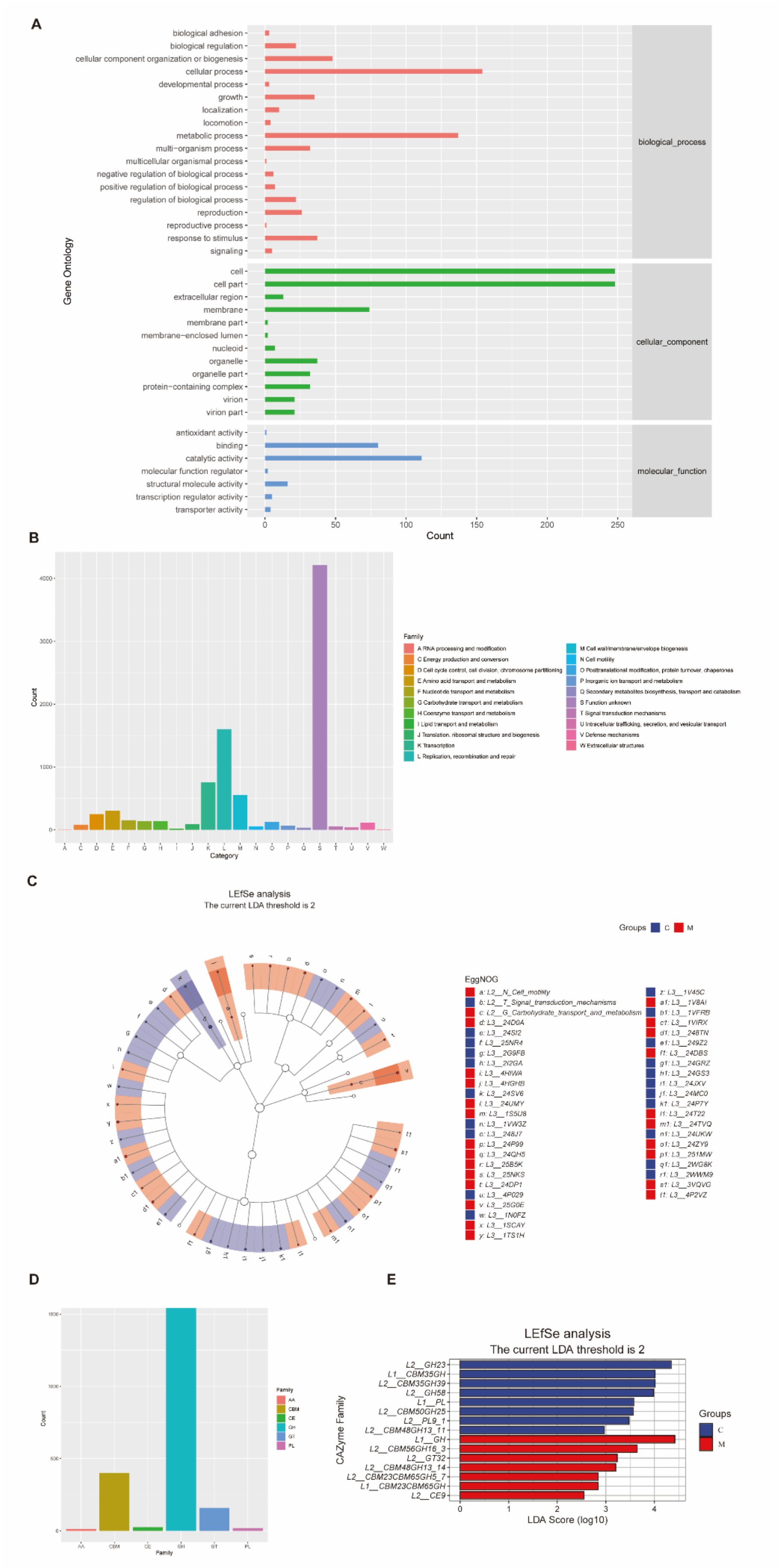

A
Gene Ontology
biological adhesion
biological regulation
cellular component organization or biogenesis
cellular process
developmental process
growth
localization
locomotion
metabolic process
multi−organism process
multicellular organismal process
negative regulation of biological process
positive regulation of biological process
regulation of biological process
reproduction
reproductive process
response to stimulus
signaling
biological_process
cell
cell part
extracellular region
membrane
membrane part
membrane−enclosed lumen
nucleoid
organelle
organelle part
protein−containing complex
virion
virion part
cellular_component
antioxidant activity
binding
catalytic activity
molecular function regulator
structural molecule activity
transcription regulator activity
transporter activity
molecular_function
0
50
100
150
200
250
Count
B
Count
Category
Family
A RNA processing and modification
C Energy production and conversion
D Cell cycle control, cell division, chromosome partitioning
E Amino acid transport and metabolism
F Nucleotide transport and metabolism
G Carbohydrate transport and metabolism
H Coenzyme transport and metabolism
I Lipid transport and metabolism
J Translation, ribosomal structure and biogenesis
K Transcription
L Replication, recombination and repair
M Cell wall/membrane/envelope biogenesis
N Cell motility
O Posttranslational modification, protein turnover, chaperones
P Inorganic ion transport and metabolism
Q Secondary metabolites biosynthesis, transport and catabolism
S Function unknown
T Signal transduction mechanisms
U Intracellular trafficking, secretion, and vesicular transport
V Defense mechanisms
W Extracellular structures
C
LEfSe analysis
The current LDA threshold is 2
Groups C M
EggNOG
a: L2__N_Cell_motility
b: L2__T_Signal_transduction_mechanisms
c: L2__G_Carbohydrate_transport_and_metabolism
d: L3__24D0A
e: L3__24SI2
f: L3__25NR4
g: L3__2G9FB
h: L3__2I2GA
i: L3__4HIWA
j: L3__4HGHB
k: L3__24SV6
l: L3__24UMY
m: L3__1S5U8
n: L3__1VW3Z
o: L3__248J7
p: L3__24P99
q: L3__24QH5
r: L3__25B5K
s: L3__25NKS
t: L3__24DP1
u: L3__4P029
v: L3__25G0E
w: L3__1N0FZ
x: L3__1SCAY
y: L3__1TS1H
z: L3__1V45C
a1: L3__1V8AI
b1: L3__1VFRB
c1: L3__1VIRX
d1: L3__248TN
e1: L3__249Z2
f1: L3__24DBS
g1: L3__24GRZ
h1: L3__24GS3
i1: L3__24JXV
j1: L3__24MC0
k1: L3__24P7Y
l1: L3__24T22
m1: L3__24TVQ
n1: L3__24UKW
o1: L3__24ZY9
p1: L3__251MW
q1: L3__2WG8K
r1: L3__2WWM9
s1: L3__3VQVG
t1: L3__4P2VZ
D
Count
Family
AA
CBM
CE
GH
GT
PL
E
LEfSe analysis
The current LDA threshold is 2
CAZyme Family
L2__GH23
L1__CBM35GH
L2__CBM35GH39
L2__GH58
L1__PL
L2__CBM50GH25
L2__PL9_1
L2__CBM48GH13_11
L1__GH
L2__CBM56GH16_3
L2__GT32
L2__CBM48GH13_14
L2__CBM23CBM65GH5_7
L1__CBM23CBM65GH
L2__CE9
0
1
2
3
4
LDA Score (log10)
Groups
C
M

Supplementary Figure 2. Extended functional annotation of the gut virome.

(A) Gene Ontology (GO) classification of predicted viral genes.

(B) EggNOG functional classification.

(C) LEfSe (LDA > 2.0) of EggNOG functions.

(D) CAZy family distribution.

(E) LEfSe (LDA > 2.0) of CAZy families. C, Control group; M, model group.

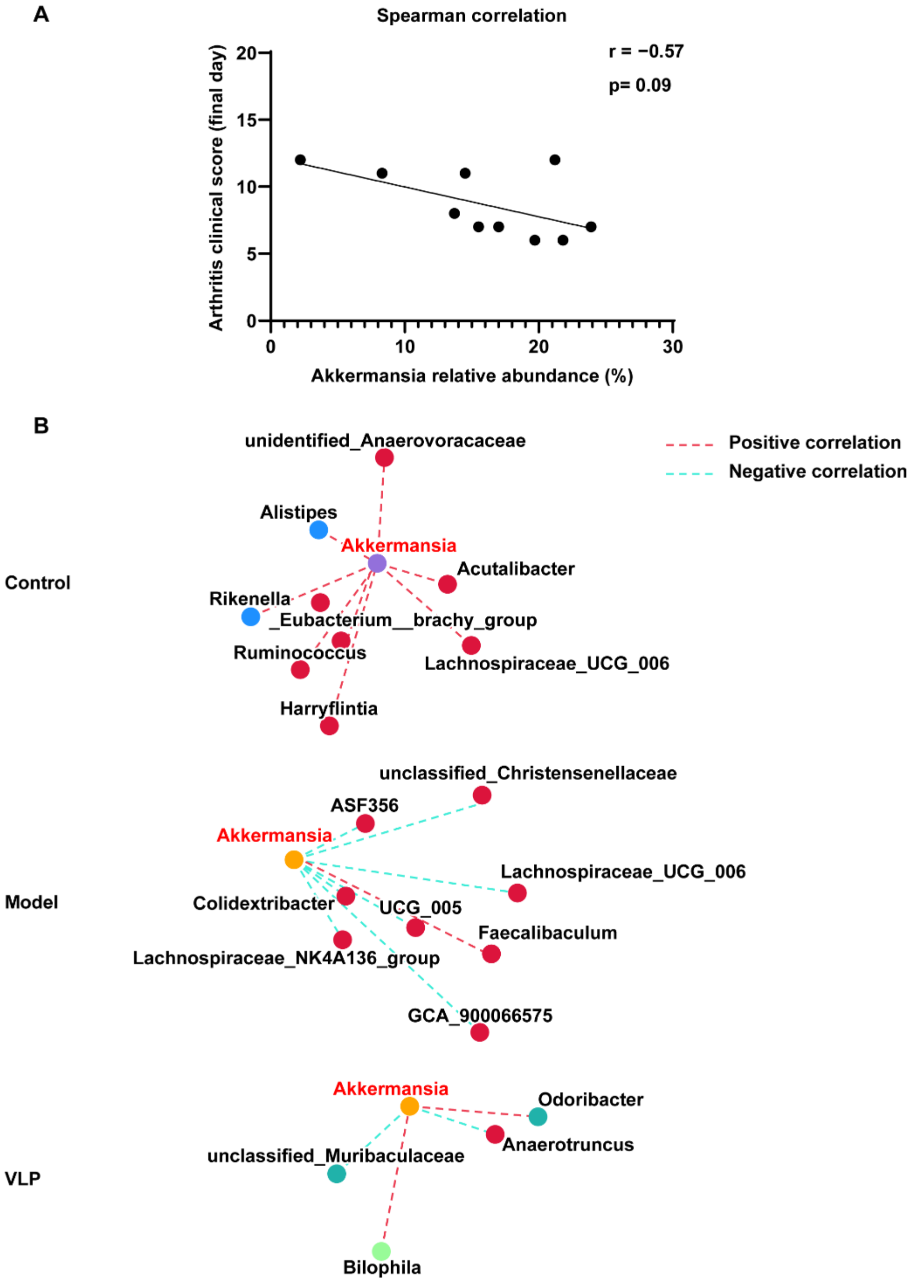

Supplementary Figure 3. Correlation analysis of Akkermansia abundance and microbial

association networks.

(A) Spearman correlation between Akkermansia relative abundance and arthritis clinical scores.

(B) Genus-level microbial correlation networks centered on Akkermansia in control, model, and VLP groups. Red dashed lines indicate positive correlations and blue dashed lines indicate negative correlations.

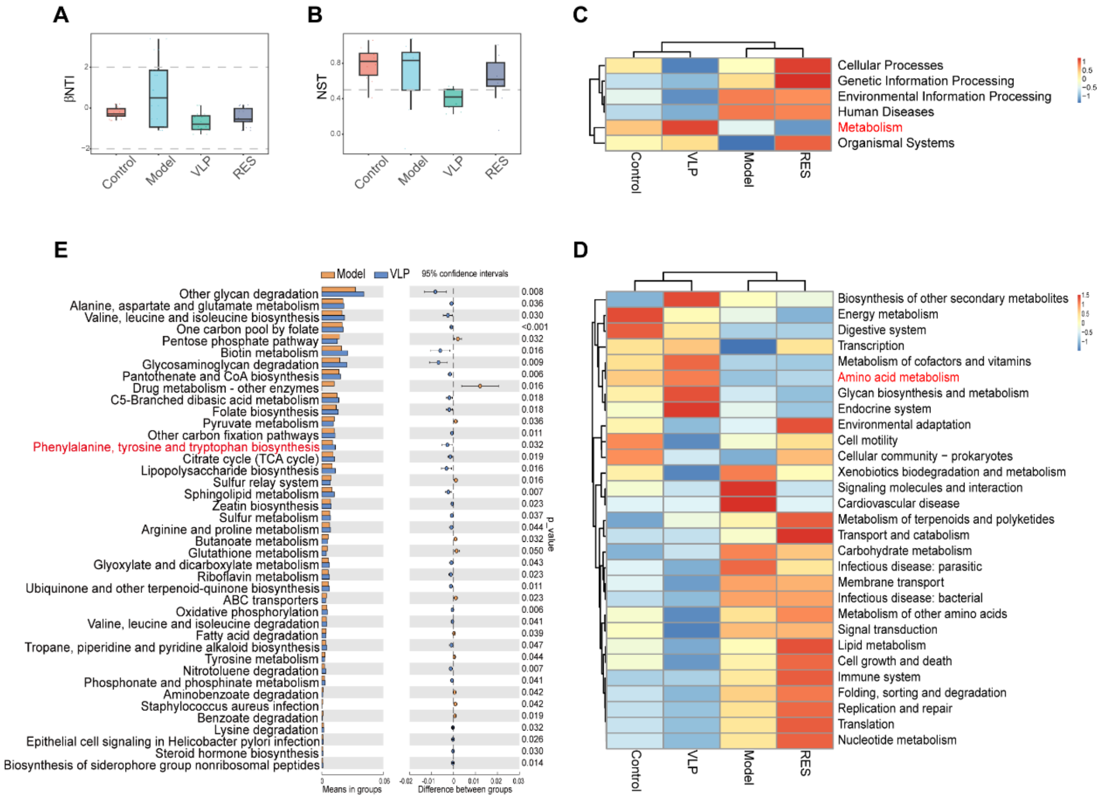

Supplementary Figure 4. Ecological assembly processes and predicted functional alterations of gut microbiota following FVT.

(A) βNTI distribution across groups.

(B) Normalized stochasticity ratio (NST) indicating relative contributions of deterministic and stochastic processes in microbial community assembly.

(C) Heatmap showing KEGG level 1 functional categories.

(D) Heatmap showing KEGG level 2 functional pathways.

(E) Differential KEGG pathways between Model and VLP groups identified by statistical comparison.
