# Supplementary figures and images for: Fecal virome transplantation attenuates arthritis in mice by remodeling gut ecology, systemic tryptophan metabolism, and innate immune responses
Source: NPJ Biofilms Microbiomes. 2026 Apr 8;12:111. doi: 10.1038/s41522-026-00980-2 (PMC13236970; doi:10.1038/s41522-026-00980-2)

Supplementary figure. Uncropped Gels for Figure 4.

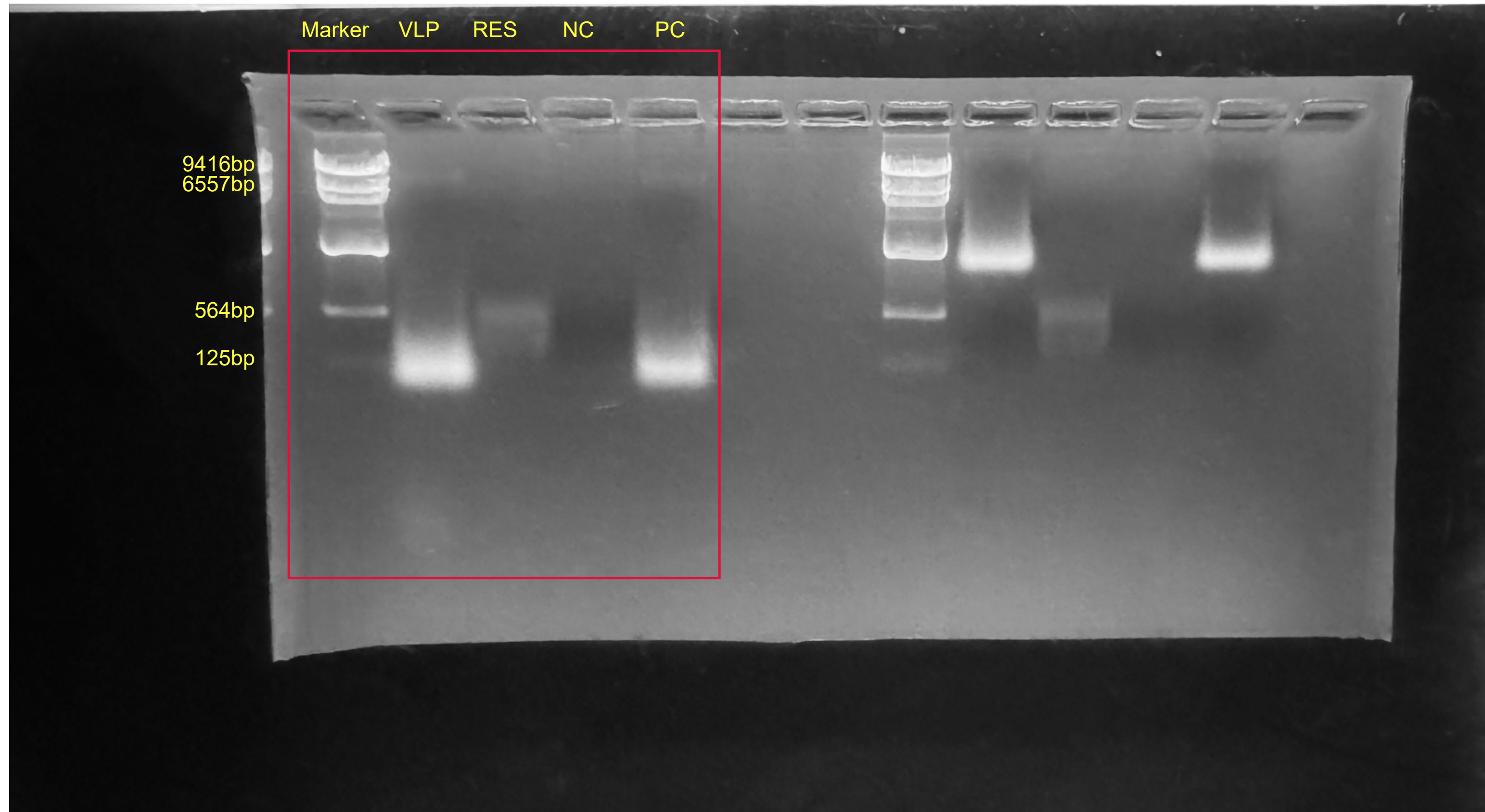

Supplement: Supplementary file 2 — 41522_2026_980_MOESM2_ESM [file 41522_2026_980_MOESM2_ESM.pdf]
